# Posicionamento para Ressuscitação Cardiopulmonar de Pacientes com Diagnóstico ou Suspeita de COVID-19 – 2020

**DOI:** 10.36660/abc.20200548

**Published:** 2020-06-29

**Authors:** Hélio Penna Guimarães, Sérgio Timerman, Roseny dos Reis Rodrigues, Thiago Domingos Corrêa, Daniel Ujakow Correa Schubert, Ana Paula Freitas, Álvaro Rea, Thatiane Facholi Polastri, Matheus Fachini Vane, Thomaz Bittencourt Couto, Antonio Carlos Aguiar Brandão, Natali Schiavo Giannetti, Thiago Timerman, Ludhmila Abrahão Hajjar, Fernando Bacal, Marcelo Antônio Cartaxo Queiroga Lopes

**Affiliations:** 1 Hospital Israelita Albert Einstein São PauloSP Brasil Hospital Israelita Albert Einstein , São Paulo , SP – Brasil; 2 Hospital das Clínicas Faculdade de Medicina Universidade de São Paulo São PauloSP Brasil Instituto do Coração do Hospital das Clínicas da Faculdade de Medicina da Universidade de São Paulo , São Paulo , SP – Brasil; 3 Hospital Estadual Getúlio Vargas Rio de JaneiroRJ Brasil Hospital Estadual Getúlio Vargas , Rio de Janeiro RJ – Brasil; 4 Hospital de Pronto Socorro de Porto Alegre RS Brasil Hospital de Pronto Socorro de Porto Alegre , RS – Brasil; 5 Universidade Federal do Paraná CuritibaPR Brasil Universidade Federal do Paraná , Curitiba , PR – Brasil; 6 Hospital das Clínicas Faculdade de Medicina Universidade de São Paulo São PauloSP Brasil Hospital das Clínicas da Faculdade de Medicina da Universidade de São Paulo , São Paulo , SP – Brasil; 7 Faculdade de Ciências Médicas de São José dos Campos HUMANITAS São José dos CamposSP Brasil Faculdade de Ciências Médicas de São José dos Campos – HUMANITAS , São José dos Campos , SP – Brasil; 8 Universidade do Vale do Sapucaí Pouso AlegreMG Brasil Universidade do Vale do Sapucaí , Pouso Alegre , MG – Brasil; 9 Hospital das Clínicas Faculdade de Medicina Universidade de São Paulo São PauloSP Brasil Instituto da Criança e do Adolescente do Hospital das Clínicas da Faculdade de Medicina da Universidade de São Paulo , São Paulo , SP – Brasil; 10 Hospital Sancta Maggiore São PauloSP Brasil Hospital Sancta Maggiore , São Paulo , SP – Brasil; 11 Faculdade de Medicina Universidade de São Paulo São PauloSP Brasil Faculdade de Medicina da Universidade de São Paulo , São Paulo , SP – Brasil; 12 Instituto do Câncer do Estado de São Paulo São PauloSP Brasil Instituto do Câncer do Estado de São Paulo , São Paulo , SP – Brasil; 13 Hospital Alberto Urquiza Wanderley João PessoaPB Brasil Hospital Alberto Urquiza Wanderley , João Pessoa , PB – Brasil

## Abstract

A atenção ao paciente vítima de parada cardiorrespiratória no contexto da pandemia da Doença por Coronavírus 2019 (COVID-19) possui particularidades que devem ser ressaltadas. As seguintes recomendações da Associação Brasileira de Medicina de Emergência (ABRAMEDE), Sociedade Brasileira de Cardiologia (SBC), Associação de Medicina Intensiva Brasileira (AMIB) e Sociedade Brasileira de Anestesiologia (SBA), representantes oficiais de especialidades afiliadas à Associação Médica Brasileira (AMB), têm por objetivo orientar as diversas equipes assistentes, em uma situação de poucas evidências sólidas, maximizando a proteção das equipes e dos pacientes.

É fundamental a paramentação completa com Equipamentos de Proteção Individual para aerossóis durante o atendimento de parada cardiorrespiratória. Também se faz imperativo que se considerem e tratem as potenciais causas nesses pacientes, principalmente hipóxia e arritmias de correntes de QT longo ou miocardite. A instalação de via aérea invasiva avançada deve ser obtida precocemente, e o uso de filtros HEPA na interface com a bolsa-válvula é obrigatório. Situações de ocorrência de parada cardiorrespiratória durante a ventilação mecânica e em posição pronada demandam peculiaridades de ajustes do ventilador e posicionamento de compressões torácicas distintas do padrão de ressuscitação cardiopulmonar. Dadas essas particularidades logísticas, o atendimento segue de acordo com os protocolos e diretrizes nacionais e internacionais do *International Liaison Committee on Resuscitation* (ILCOR) de 2015, das diretrizes da *American Heart Association* (AHA) de 2019 e da Atualização da Diretriz de Ressuscitação Cardiopulmonar e Cuidados de Emergência da Sociedade Brasileira de Cardiologia de 2019.

**Table:** 

Declaração de potencial conflito de interesses dos autores/colaboradores do Posicionamento para Ressuscitação Cardiopulmonar de Pacientes com Diagnóstico ou Suspeita de COVID-19 – 2020							
Se nos últimos 3 anos o autor/colaborador do Posicionamento:							
Nomes Integrantes do Posicionamento	Participou de estudos clínicos e/ou experimentais subvencionados pela indústria farmacêutica ou de equipamentos relacionados à diretriz em questão	Foi palestrante em eventos ou atividades patrocinadas pela indústria relacionados à diretriz em questão	Foi (é) membro do conselho consultivo ou diretivo da indústria farmacêutica ou de equipamentos	Participou de comitês normativos de estudos científicos patrocinados pela indústria	Recebeu auxílio pessoal ou institucional da indústria	Elaborou textos científicos em periódicos patrocinados pela indústria	Tem ações da indústria
Álvaro Rea Neto	Não	Não	Não	Não	Não	Não	Não
Ana Paula Freitas	Não	Não	Não	Não	Não	Não	Não
Antonio Carlos Aguiar Brandão	Não	Não	Não	Não	Não	Não	Não
Daniel Ujakow Correa Schubert	Não	Não	Não	Não	Não	Não	Não
Fernando Bacal	Não	Não	Não	Não	Não	Não	Não
Hélio Penna Guimarães	Não	Não	Não	Não	Não	Não	Não
Ludhmila Abrahão Hajjar	Não	Não	Não	Não	Não	Não	Não
Marcelo Antônio Cartaxo Queiroga Lopes	Não	Não	Não	Não	Não	Não	Não
Matheus Fachini Vane	Não	Não	Não	Não	Não	Não	Não
Natali Schiavo Giannetti	Não	Não	Não	Não	Não	Não	Não
Roseny dos Reis Rodrigues	Não	CSL Behring, Baxter, União Química, Octapharma	Halexistar	Não	Não	Octapharma	Não
Sérgio Timerman	Não	Não	Não	Não	Não	Não	Não
Thatiane Facholi Polastri	Não	Não	Não	Não	Não	Não	Não
Thiago Domingos Correa	Não	Não	Não	Não	Não	Não	Não
Thiago Timerman	Não	Não	Não	Não	Não	Não	Não
Thomaz Bittencourt Couto	Não	Não	Não	Não	Não	Não	Não

## 1. Introdução

A Ressuscitação Cardiopulmonar (RCP) é um procedimento máximo de emergência e passível de ocorrência em pacientes portadores de Doença por Coronavírus 2019 (COVID-19). Demanda, assim, atenção especial, particularmente quanto ao risco maior de aerossóis durante as manobras de compressão torácica e ventilação, oferecendo risco relevante de contaminação para a equipe.

Considerando esse cenário, em que evidências sólidas estão pouco documentadas ou acessíveis, a Associação Brasileira de Medicina de Emergência (ABRAMEDE), a Sociedade Brasileira de Cardiologia (SBC), a Associação de Medicina Intensiva Brasileira (AMIB) e a Sociedade Brasileira de Anestesiologia (SBA), representantes oficiais de especialidades afiliadas à Associação Médica Brasileira (AMB), apresentam, a seguir, as práticas destinadas especificamente ao atendimento de pacientes com diagnóstico ou suspeita de COVID-19. Em todos os outros casos, mantêm-se as diretrizes de 2015 da International Alliance of Resuscitation Committees (ILCOR), as diretrizes de 2019 da *American Heart Association* (AHA) ^1^ e a Atualização da Diretriz de Ressuscitação Cardiopulmonar e Cuidados de Emergência da Sociedade Brasileira de Cardiologia de 2019. ^2^

## 2. Prevenção da Parada Cardiorrespiratória

- Todos os pacientes suspeitos ou portadores de COVID-19, que estejam sob maior risco de deterioração aguda ou Parada Cardiorrespiratória (PCR), devem ser adequadamente sinalizados aos Times de Resposta Rápida (TRR) ou equipes que procederão ao atendimento. ^3 - 5^ O uso de escores de gravidade e sistemas de rastreamento e o disparo de códigos amarelos permitem a detecção precoce de pacientes graves e podem otimizar o atendimento de eventuais PCRs; ^2 , 5^
- A avaliação de potencial dificuldade para laringoscopia/intubação deve ser realizada na admissão do paciente no hospital e/ou em Unidades de Terapia Intensiva (UTI) e estar registrada em prontuário. Escores como MACOCHA ou mnemônicos como LEMON (de *Look-Evaluate-Mallampati-Obstruction-Neck* ) podem auxiliar na determinação de via aérea difícil, no prévio acionamento de suporte e na solicitação de equipamentos de via aérea difícil; ^6 , 7^
- Considerando as recentes terapias em fase de avaliação com cloroquina ou hidroxicloroquina e seu potencial risco para alargar o intervalo QT em até 17% dos casos, é fundamental considerar o risco de arritmias ventriculares polimórficas graves, especialmente *torsades de pointes* , e consequente ocorrência de PCR em ritmos chocáveis; ^4 , 8 - 10^
- Os pacientes de maior risco para taquicardias polimórficas nesse contexto são os idosos; do sexo feminino; com miocardite relacionada à COVID, insuficiência cardíaca, disfunção hepática ou renal, distúrbios eletrolíticos (particularmente redução de potássio e magnésio) e bradicardia. É fundamental identificar os pacientes que já tenham intervalo QT corrigido (QTc) prolongado (superior a 500 ms) com monitoração diária do Eletrocardiograma (ECG) durante o uso dos fármacos. ^4 , 8 - 10^

## 3. Tomada de Decisão

- Os processos da tomada de decisão para iniciar a RCP, ou não, devem continuar sendo individualizados nos serviços de atendimento pré-hospitalar, departamentos de emergência e UTI. Deve-se sempre levar em consideração os benefícios ao paciente, a segurança e exposição da equipe e o potencial de futilidade das manobras. A RCP deve ser sempre realizada, a menos que diretivas previamente definidas indiquem o contrário; ^1 , 2^
- As decisões/diretivas de “Não Ressuscitação Cardiopulmonar” (NRCP) devem estar adequadamente documentadas e ser comunicadas à equipe. Os cuidados paliativos e de terminalidade devem seguir a política local e institucional. ^1 , 2^

## 4. Orientações sobre Precauções

- A precaução por padrão + aerossol é a indicada para todos os membros da equipe de ressuscitação, a fim de garantir a adequada proteção individual. A pronta disponibilidade de Equipamentos de Proteção Individual (EPIs), como *kits* de paramentação no carro de emergência, promove menor retardo no início das compressões torácicas e continuidade do atendimento. ^3 , 4 , 11 - 14^ Devem constar no *kit* de EPI máscara N95, *face shield,* avental impermeável, gorro, luvas descartáveis de cano alto e óculos de proteção;
- Ainda que possa ocorrer atraso no início das compressões torácicas, a segurança da equipe é prioritária, e o uso de EPIs adequados é indispensável para os que atendem à PCR. Em particular, não se deve iniciar a RCP em um paciente suspeito ou confirmado de COVID-19 até que a equipe esteja totalmente paramentada; ^3 , 4 , 11 - 14^
- Restrinja o número de funcionários no local do atendimento (se for um quarto individual comum); ^2 , 4 , 15 , 16^
- A higiene das mãos tem papel importante na redução da transmissão da COVID-19. Higienize as mãos adequadamente com água e sabão, em caso de sujidade, ou álcool em gel; ^3 , 15^
- É importante que todas as orientações do Ministério da Saúde e dos governos locais sejam adequadamente respeitadas.

## 5. Atendimento Inicial

- O reconhecimento da PCR segue a conduta preconizada pelo ILCOR/AHA e pela SBC, sendo iniciada por avaliação da responsividade, respiração (somente avaliação dos movimentos respiratórios) e presença de pulso central; ^1 , 2^
- A RCP deve ser iniciada por compressões torácicas contínuas em adultos. Se o paciente ainda não estiver com uma via aérea invasiva/avançada instalada (tubo orotraqueal ou dispositivo extraglótico), deve manter-se a máscara de oxigênio, com baixo fluxo ou uma toalha sobre a boca e nariz do mesmo, até que a via aérea invasiva seja obtida, ^8^ já que movimentos de compressão torácica podem desencadear eliminação de aerossóis; devendo ser iniciados com atenção a esse cuidado descrito;
- Em crianças, fazer preferencialmente RCP com compressões e ventilações com bolsa-válvula-máscara (BVM) acoplada ao filtro *High Efficiency Particulate Arrestance* (HEPA), até obtenção da via aérea definitiva, uma vez que a parada pediátrica ocorre, na maioria das vezes, secundária a causas respiratórias, e a RCP somente com compressão é sabidamente menos eficaz nessa população. ^3^ Caso o equipamento não esteja disponível, uma alternativa razoável é a RCP somente com compressão, mantendo o paciente com máscara ou toalha sobre a boca; ^17^
- Mesmo com a orientação de alguns serviços para que os cuidados de atendimento pré-hospitalar à PCR, na ausência de um profissional médico, sejam realizados com RCP somente com as mãos *(hands-only)* , o cuidado descrito relativo à vedação da cavidade oral do paciente para proteção de aerossolização permanece recomendado; ^4 , 8 , 9 , 14^
- A monitorização para determinação do ritmo/modalidade de parada (chocável ou não chocável) deve ser realizada o mais rápido possível, para não atrasar a desfibrilação de um ritmo chocável e o estabelecimento do algoritmo adequado; ^1 , 2^
- A desfibrilação em ritmos chocáveis não deve ser adiada para acesso às vias aéreas ou outros procedimentos; ^1 , 2^
- Se o paciente estiver com máscara facial de oxigenação antes da ocorrência da PCR, mantenha a mesma até a intubação, porém sem alto fluxo de oxigênio (6 a 10 L/minuto no máximo), pois, do contrário, sobe o risco de geração de aerossol;
- Se o paciente não estiver com dispositivo de via aérea, o profissional deve colocar um pano/toalha sobre a boca e o nariz da vítima e, então, realizar compressões contínuas;
- Identifique e trate quaisquer causas reversíveis antes de considerar interromper a RCP, com especial consideração para hipóxia, acidemia e trombose coronária, causas citadas como frequentes nas publicações atuais sobre COVID-19. ^3^ Adicionalmente, taquicardia ventricular polimórfica do tipo *torsade de pointes* (associada ao alargamento de QT desencadeado por fármacos em tratamento) e tamponamento cardíaco (associada à miocardite), bem como eventual pneumotórax associado à ventilação mecânica, estão descritos como causas de PCR.

## 6. Manejo das Vias Aéreas

- Deve-se evitar a ventilação com BVM ou bolsa-tubo endotraqueal, pelo elevado risco de gerar aerossóis e contaminação da equipe. ^3 , 15 , 18 , 19^ No caso de absoluta necessidade de ventilação com BVM, a técnica de selamento da máscara deve sempre envolver dois profissionais, e deve-se utilizar uma cânula orofaríngea (Guedel). Nesse caso, realizam-se 30 compressões e duas ventilações, em adultos, e 15 compressões e duas ventilações, em crianças, até que a via aérea invasiva seja estabelecida, quando se recomenda uma ventilação a cada 6 segundos para adultos e crianças. Preconiza-se a instalação de filtros HEPA entre a máscara e a bolsa ( Figuras 1 a 3 );
  **Figura 1:**
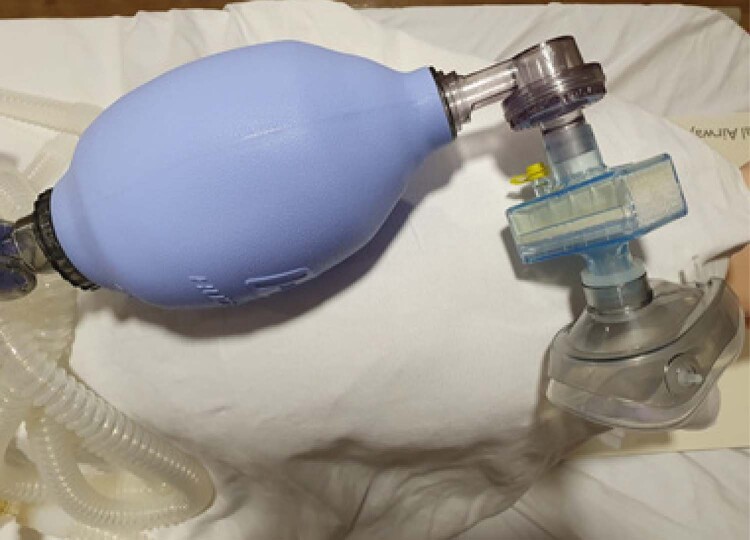
– Dispositivo bolsa-válvula-máscara com filtro HEPA. Fonte: Arquivo pessoal dos autores.
- Considerando ser a hipóxia uma das principais causas de PCR nesses pacientes, o acesso invasivo da via aérea deve ser priorizado, para isolamento, por menor probabilidade de geração de aerossóis e consequente menor contaminação da equipe, bem como melhor padrão de ventilação/oxigenação. ^15 , 16 , 19 - 21^ Durante a instrumentalização da via aérea, a compressão torácica deve ser interrompida para proteção da equipe. Sugere-se que sua instrumentalização ocorra nos períodos de checagem de pulso, para diminuir o intervalo sem compressões, e recomenda-se que o médico mais experiente realize a intubação orotraqueal;
- A intubação com uso de videolaringoscopia com lâmina de maior angulação deve ser a primeira escolha para o acesso rápido, seguro e definitivo às vias aéreas, sendo prioridade desde a primeira tentativa, realizada sempre pelo médico mais experiente. No caso de falha, a ajuda/apoio de um segundo médico deve imediatamente ser solicitada, que, em segunda tentativa, deve novamente priorizar o uso da videolaringoscopia; ^16 , 20 , 21^
- Para crianças, recomenda-se videolaringoscopia com lâmina adequada ao tamanho do paciente, sem necessidade de maior angulação; ^20^
- Na impossibilidade ou falha na intubação, devem-se utilizar dispositivos extraglóticos (tubo laríngeo ou máscara laríngea), que permitam a ventilação mecânica em circuito fechado, além do uso de capnografia, até que haja a adequada possibilidade de acesso definitivo à via aérea (intubação traqueal ou cricostomia). ^20 , 22^ Em crianças, utilizar preferencialmente máscara laríngea adequada ao peso como dispositivo extraglótico. ^23^ No Brasil, a instalação de dispositivos extraglóticos faz parte do escopo profissional de médicos e enfermeiros, podendo ser alternativa para o acesso às vias aéreas em unidades de suporte intermediário pré-hospitalar e nos atendimentos realizados por enfermeiros. ^1 , 2^ No entanto, recomenda-se a utilização do tubo endotraqueal sempre que possível, com o objetivo de reduzir a formação de aerossol;
- Quanto aos dispositivos extraglóticos, dentre os disponíveis, deve-se priorizar, sempre que possível, os com maior vedação e que apresentam a possibilidade de sequencial introdução do tubo orotraqueal através dele ( *fast track* );
- Mesmo intubado ou com dispositivo extraglótico, são importantes a oclusão e a vedação da boca do paciente, o que pode ser realizado com toalhas, gazes ou máscaras cirúrgicas, para reduzir a aerossolização.
- Quando a PCR ocorrer em pacientes sob ventilação mecânica, deve-se manter o paciente conectado ao ventilador em circuito de ventilação fechado e ajustar os parâmetros da seguinte forma ( Quadro 1 ):
  **Quadro 1:** – Passos para setar o ventilador mecânico para ressuscitação cardiopulmonar.

Modo assistido-controlado a volume ≥ 6 mL/kg peso predito Frequência respiratória ≥ 10 irpm FiO _2_ 100% Disparo (trigger) a fluxo: desligar a sensibilidade ou -15 a -20 PEEP = 0 Alarmes de volume corrente máximo e mínimo permitidos pelo equipamento Alarmes de pressão máxima de 60 cmH _2_ O e mínima de 1 ou 0 cmH _2_ O Alarmes de volume-minuto máximo e mínimo de cada aparelho Alarme de tempo de apneia de 60 segundos

FiO _2_: fração inspirada de oxigênio; PEEP: pressão positiva expiratória final.

Fonte: Arquivo dos autores.
  ○ Modo a volume, assistido-controlado, ajustado a 6 mL/kg do peso predito do paciente;
  ○ Fração inspirada de oxigênio a 100%;
  ○ Frequência respiratória em torno de dez ventilações por minuto e tempo inspiratório de 1 segundo;
  ○ Disparo ( *trigger* ) a fluxo: desligar a sensibilidade; caso impossível, mudar o modo a pressão da sensibilidade e ajustá-la para a forma menos sensível possível (varia de acordo com ventilador de -15 a -20). A sensibilidade deve ser ajustada para o modo menos sensível ao disparo;
  ○ Pressão positiva no final da expiração (PEEP) de zero;
  ○ Sobre os alarmes, ajustar para alarmes de volume corrente máximo e mínimo permitidos pelo equipamento, e alarmes de pressão máxima de 60 cmH _2_ O e mínima de 1 ou 0 cmH _2_ O. Alarmes de volume-minuto devem permitir o máximo e o mínimo de cada aparelho. O alarme de frequência respiratória deve ser ajustado para o máximo permitido e o tempo de apneia deve ser de 60 segundos.

- Os mesmos parâmetros devem ser ajustados em crianças. Avalie continuamente se o ventilador consegue manter esses parâmetros sem autodisparo pela compressão, gerando hiperventilação e aprisionamento de ar, com pressões excessivas (sistematicamente acima de 60 cmH _2_ O). Em crianças, pode ser necessário desconectar do ventilador; neste caso, deve-se utilizar bolsa-válvula conectado a filtro HEPA;
- Alguns ventiladores apresentam a função “RCP/PCR”, que ajusta automaticamente os limites de alarme e aciona os parâmetros alinhados acima. Em ventilação mecânica, preconiza-se a instalação de filtros HEPA no circuito ventilatório após o tubo orotraqueal, e outro na via do circuito expiratório; ^16 , 20 , 21^
- Pinças retas fortes podem ser usadas para clampear o tubo, quando houver necessidade de mudança de circuitos/ventiladores (bolsa-válvula-máscara para o circuito de ventilador mecânico, por exemplo), com o objetivo de minimizar a aerossolização;
- Ao aplicar a desfibrilação, para segurança da equipe e do paciente, deve-se sempre preferir o uso de pás adesivas, que não demandem a necessidade de desconexão do ventilador para liberação do choque. Caso sejam usadas pás manuais para desfibrilação, deve-se liberar o choque; após, colocar o ventilador em modo *stand-by* e desconectar o tubo orotraqueal do ventilador, mantendo o filtro HEPA acoplado ao tubo.

## 7. Compressões Torácicas

- Realizar as compressões torácicas de alta qualidade, garantindo:
  ○ Frequência das compressões de 100 a 120 compressões por minuto;
  ○ Em adultos, profundidade de, no mínimo, 5 cm (evitando compressões com profundidade maior que 6 cm);
  ○ Em lactentes, profundidade de um terço do diâmetro anteroposterior do tórax e, em crianças, um terço do diâmetro anteroposterior do tórax ou no mínimo 5 cm.

- Permitir o retorno completo do tórax após cada compressão, evitando apoiar-se no tórax da vítima;
- Minimizar interrupções das compressões; pause no máximo 10 segundos para realização de duas ventilações. Considere obter uma fração de compressão torácica a maior possível, tendo como objetivo o mínimo de 60% a 80%;
- Revezar com outro socorrista a cada 2 minutos, para evitar cansaço e compressões de má qualidade;
- Se o paciente estiver em decúbito dorsal horizontal, realizar as compressões no centro do tórax, na metade inferior do osso esterno;
- Entendendo particularidades do uso de EPIs para aerossolização pelos profissionais, a alta demanda física das manobras, seu potencial de exaustão, e a necessidade de minimização da equipe presente na ressuscitação, sugere-se o uso de dispositivos mecânicos de RCP para adultos, caso disponíveis.

## 8. Ressuscitação em Posição Pronada/Prona

- Caso o paciente esteja em posição pronada, sem via aérea invasiva instalada, recomenda-se reposicioná-lo rapidamente em posição supina, estabelecer as manobras de RCP e, o mais breve possível, instalar a via aérea invasiva, preferencialmente por intubação orotraqueal;
- Caso o paciente já esteja sob intubação orotraqueal e ventilação mecânica, recomenda-se iniciar as manobras de RCP com o paciente ainda em posição prona. O ponto de referência para posicionamento das mãos segue a projeção do mesmo lugar das compressões torácicas (T7-T10), na região interescapular ( Figura 4 ). Recomenda-se que tentativas de retorno do paciente para posição supina sejam executadas com o máximo de segurança ao despronar, evitando a desconexão do ventilador e o risco de aerossolização. Se houver disponibilidade de pás adesivas do desfibrilador, deve–se colá-las em posição anteroposterior; ^10 , 22 , 23^
  **Figura 4:**
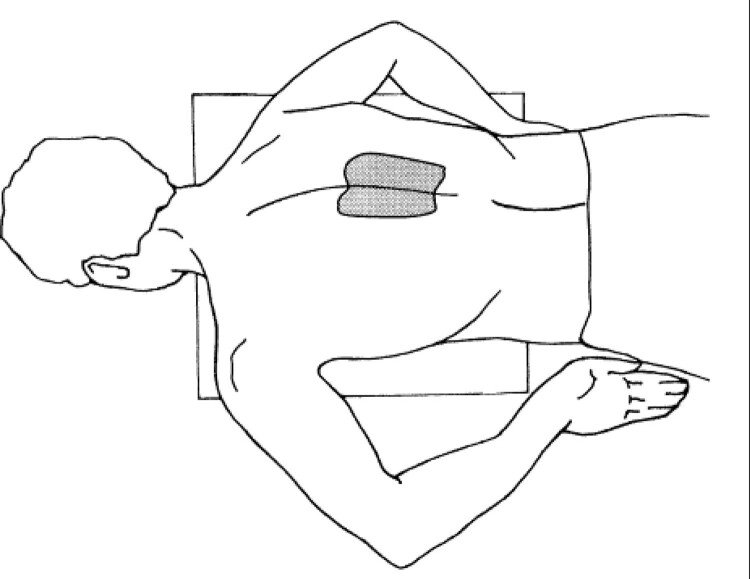
– Local das mãos para realização das compressões em pacientes em posição prona. 23
- Caso não existam pás adesivas, a desfibrilação deve ser tentada colocando-se a pá esternal na região dorsal e a pá apical na lateral do paciente ( Figura 5 ). Recomenda-se que a eficácia da RCP seja avaliada usando dióxido de carbono expirado (pressão parcial de dióxido de carbono > 10 mmHg) e pressão arterial invasiva (considerando valores da pressão diastólica > 20 mmHg). Convém citar que as evidências para esta manobra são ainda incertas e, sempre que possível, a reversão da posição prona para a supina (mais adequada para realização da RCP de alta qualidade, bem como adequada ventilação) deve ser realizada.
  **Figura 5:**
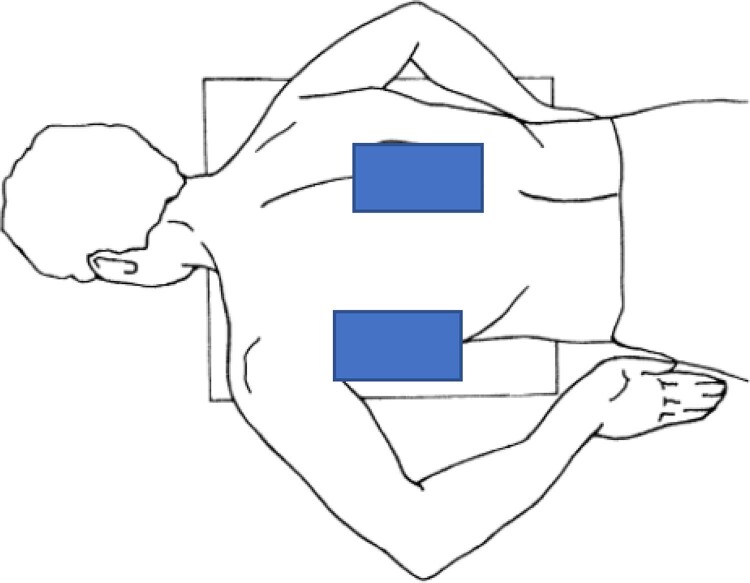
– Posição sugerida das pás para desfibrilação em pacientes em posição prona. 23

## 9. Pós-parada Cardiorrespiratória

- Antecipe a solicitação de leito em UTI com isolamento respiratório, antes do paciente ter o Retorno à Circulação Espontânea (RCE); ^3 , 15 , 16^
- Descarte ou higienize todo o equipamento usado durante a RCP, seguindo as recomendações do fabricante e as diretrizes locais da instituição; ^3^
- Quaisquer superfícies usadas para posicionar equipamentos de vias aéreas/ressuscitação também precisam ser higienizadas, de acordo com as diretrizes locais. Verifique se o equipamento usado no manejo das vias aéreas (por exemplo, laringoscópio e máscaras faciais) não foi deixado sobre o leito do paciente. Procure deixar os equipamentos em uma bandeja; ^3 , 18^
- Após o atendimento, remova o EPI com segurança, evitando a autocontaminação. ^3 , 15^ Para esse passo, deve ser dada toda atenção, considerando que a maior parte da contaminação dos profissionais de saúde ocorre nesse momento, por contato com secreções e gotículas.

## 10. Orientações Para Ambiente Pré-hospitalar

- Em ambiente pré-hospitalar, não deve ser iniciada a RCP em pacientes suspeitos ou confirmados com COVID-19, com sinais óbvios de morte; ^3^
- Os profissionais devem utilizar precaução padrão + aerossol para o atendimento de vítimas suspeitas ou confirmadas com COVID-19;
- Orientar a população que, ao ligar 192, informar se a vítima é suspeita de COVID-19. Isso facilita a paramentação prévia da equipe de atendimento. Sugere-se que os telefonistas e reguladores do serviço médico de emergência realizem busca ativa desses pacientes, indagando sobre sintomas gripais, febre e dispneia;
- Realize compressões contínuas. A ventilação boca-boca e o uso de máscara de bolso não devem ser realizadas para pacientes suspeitos ou confirmados com COVID-19; ^3^
- Considerando que a maioria das paradas extra-hospitalares ocorre no domicílio, na PCR extra-hospitalar pediátrica, o socorrista leigo muito provavelmente é um membro da família ou cuidador da criança, que já está em contato próximo e exposto a secreções. Nesse caso, o socorrista leigo deve realizar compressões e considerar ventilação boca a boca, caso seja capaz e esteja disposto a isso, uma vez que a maioria das paradas pediátricas ocorre por causa respiratória; ^23^
- A RCP somente com compressão é alternativa razoável, caso o socorrista não se seja capaz de fazer a ventilação ou não tenha tido contato prévio próximo com a criança; ^17^
- Os socorristas devem colocar um pano ou uma toalha sobre a boca e o nariz da vítima, ou posicionar uma máscara com baixo fluxo de oxigênio contínuo, para evitar a suspensão de aerossóis durante a RCP;
- Não atrasar a desfibrilação: o uso precoce de um Desfibrilador Externo Automático (DEA) aumenta significativamente as chances de sobrevivência da pessoa e não eleva o risco de infecção;
- A ventilação com pressão positiva com BVM deve ser evitada ao máximo e, se efetivamente necessária, ser realizada por dois profissionais, sendo um deles responsável exclusivamente pelo acoplamento da máscara à face do paciente, da forma mais adequada possível, evitando vazamento de ar. A BVM só deve ser utilizada com filtro HEPA interposto à máscara;
- Em crianças, fazer preferencialmente RCP com compressões e ventilações com BVM acoplada ao filtro HEPA;
- O manejo das vias aéreas, no pré-hospitalar, deve seguir as recomendações mencionadas, de forma a garantir que as BVM e outros equipamentos de ventilação estejam equipados com filtros HEPA, e uma via área avançada (intubação orotraqueal ou dispositivo extraglótico) seja instalada precocemente;
- Abrir as portas traseiras do veículo de transporte e ativar o sistema de Aquecimento, Ventilação e Ar Condicional (AVAC) durante os procedimentos de geração de aerossóis (realizar esse procedimento longe do tráfego de pedestres);
- Não permitir que acompanhantes sejam levados na ambulância no mesmo compartimento do paciente. Os pacientes com suspeita ou confirmação de COVID-19 não podem ter acompanhantes sob risco de contaminação, segundo as recomendações do Ministério da Saúde. Sugere-se orientar que os acompanhantes se dirijam à unidade de saúde de referência por meios próprios para maiores informações;
- Se o veículo não possuir compartimento de motorista isolado, abra as saídas de ar externas na área do motorista e ligue os ventiladores de exaustão traseiros na configuração mais alta.

## 11. Treinamento e *Debriefing*

- Realize o *debriefing* ao final de cada procedimento, a fim de proporcionar melhorias e crescimento da equipe; ^1 , 2^
- Treinamento de habilidades para a correta colocação e, principalmente, a retirada do EPI e simulações de atendimento à PCR devem ser realizados o mais precocemente possível por todas as equipes envolvidas no atendimento a pacientes com suspeita ou confirmação de COVID-19; ^15 , 16 , 20 , 21^
- São imperativos o treinamento e a educação permanente, tendo em vista a proteção da equipe e a maior segurança no atendimento do paciente. Recomenda-se fortemente a utilização de cenários em ambiente de simulação realística e recursos de educação a distância.

**Figure 6:**
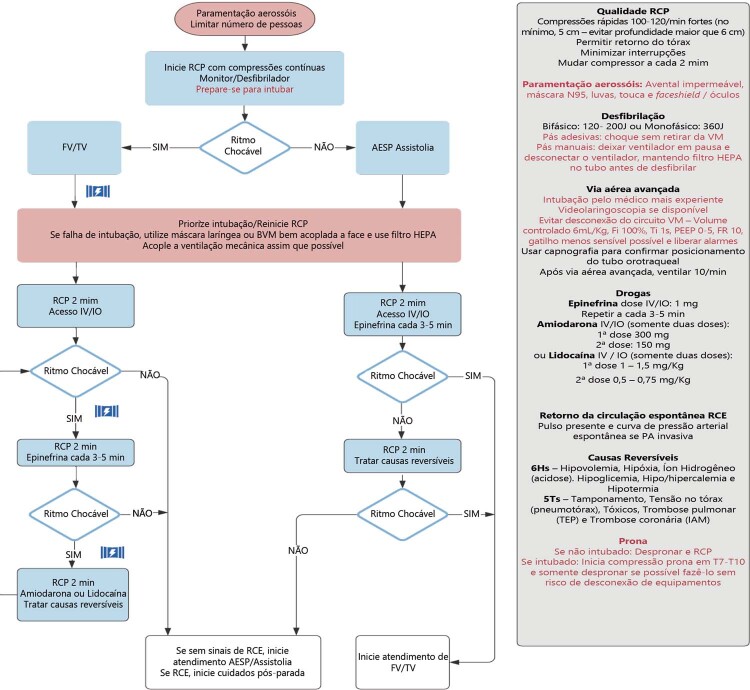
– Algoritmo de atendimento da parada cardiorrespiratória para pacientes suspeitos ou confirmados COVID-19. AESP: atividade elétrica sem pulso; BVM: bolsa-válvula-máscara; Fi: fração inspirada; FR: frequência respiratória; FV: fibrilação ventricular; HEPA: High Efficiency Particulate Arrestance; IAM: infarto agudo do miocárdio; IO: intraósseo; IV: intravenoso; PA: pressão arterial; PEEP: pressão positiva no final da expiração; RCE: retorno da circulação espontânea; RCP: ressuscitação cardiopulmonar; TEP: tromboembolismo pulmonar; Ti: tempo inspiratório; TV: taquicardia ventricular; VM: ventilação mecânica.

**Figura 7:**
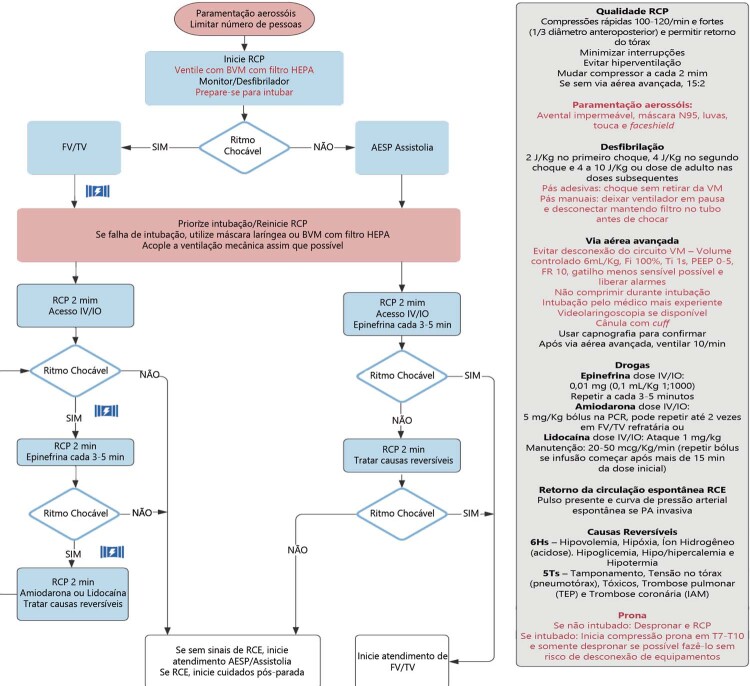
– Algoritmo de tratamento da parada cardiorrespiratória pediátrica em paciente com suspeita ou confirmação de COVID-19. AESP: atividade elétrica sem pulso; BVM: bolsa-válvula-máscara; Fi: fração inspirada; FR: frequência respiratória; FV: fibrilação ventricular; HEPA: High Efficiency Particulate Arrestance; IAM: infarto agudo do miocárdio; IO: intraósseo; IV: intravenoso; PA: pressão arterial; PEEP: pressão positiva no final da expiração; RCE: retorno da circulação espontânea; RCP: ressuscitação cardiopulmonar; TEP: tromboembolismo pulmonar; Ti: xxxx ; TV: taquicardia ventricular; VM: ventilação mecânica.

A seguir, os algoritmos para atendimento de PCR de pacientes adultos ( Figura 6 ) e pediátricos ( Figura 7 ), suspeitos ou confirmados com COVID-19.

**Figura 2:**
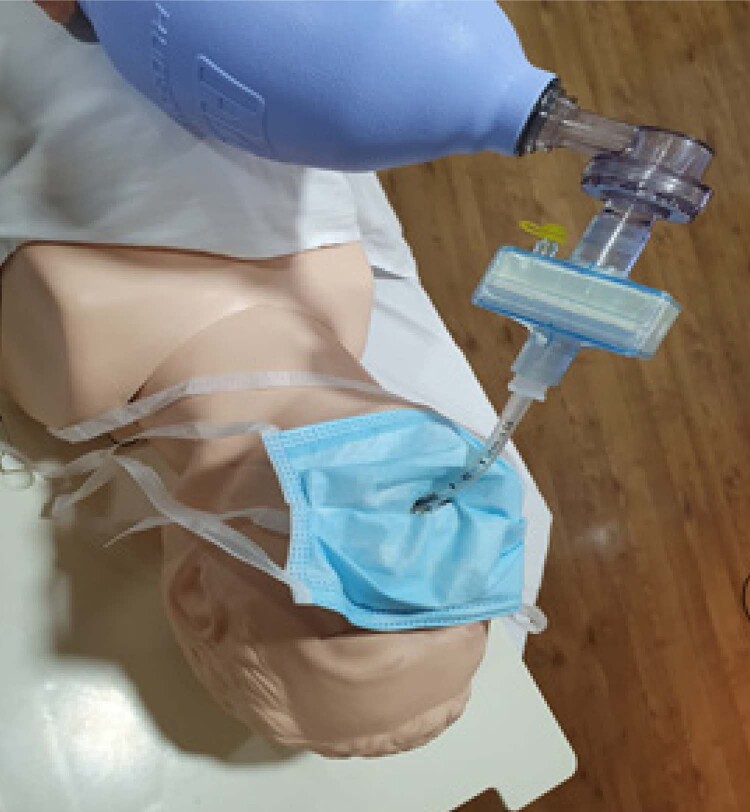
– Paciente intubado, com bolsa-válvula-máscara e filtro HEPA, além de oclusão da cavidade oral com máscara. Fonte: Arquivo pessoal dos autores.

**Figura 3:**
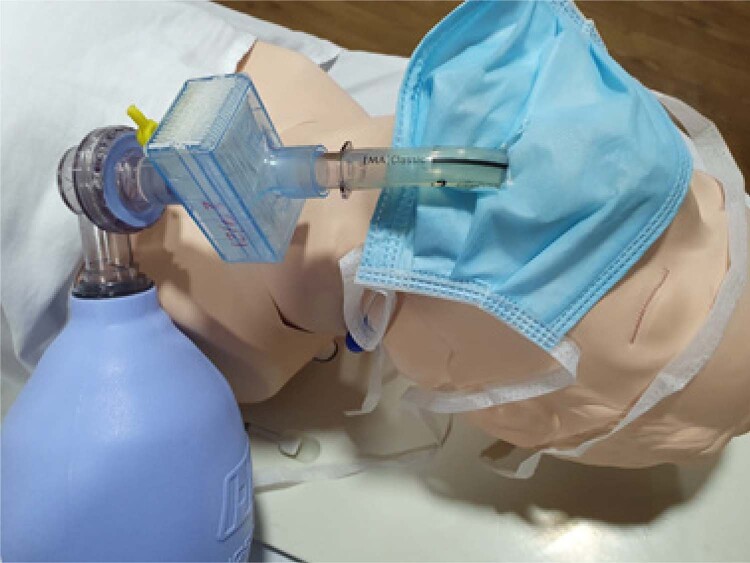
– Paciente com dispositivo extraglótico, oclusão da cavidade oral com máscara e filtro HEPA. Fonte: Arquivo pessoal dos autores.
