# Supplementary material for: Posicionamento para Ressuscitação Cardiopulmonar de Pacientes com Diagnóstico ou Suspeita de COVID-19 – 2020
Source: Arq Bras Cardiol. 2020 Jun 29;114(6):1078–87. [Article in Portuguese] doi: 10.36660/abc.20200548 (PMC8416124; doi:10.36660/abc.20200548)
Supplement: Translation

# Translation: Position Statement: Cardiopulmonary Resuscitation of Patients with Confirmed or Suspected COVID-19 – 2020

**Authors:** Hélio Penna Guimarães, Sérgio Timerman, Roseny dos Reis Rodrigues, Thiago Domingos Corrêa, Daniel Ujakow Correa Schubert, Ana Paula Freitas, Álvaro Rea, Thatiane Facholi Polastri, Matheus Fachini Vane, Thomaz Bittencourt Couto, Antonio Carlos Aguiar Brandão, Natali Schiavo Giannetti, Thiago Timerman, Ludhmila Abrahão Hajjar, Fernando Bacal, Marcelo Antônio Cartaxo Queiroga Lopes

**Affiliations:** 1 São PauloSP Brasil Hospital Israelita Albert Einstein, São Paulo , SP – Brasil; 2 São PauloSP Brasil Instituto do Coração do Hospital das Clínicas da Faculdade de Medicina da Universidade de São Paulo, São Paulo , SP – Brasil; 3 Rio de JaneiroRJ Brasil Hospital Estadual Getúlio Vargas, Rio de Janeiro RJ – Brasil; 4 RS Brasil Hospital de Pronto Socorro de Porto Alegre, RS – Brasil; 5 CuritibaPR Brasil Universidade Federal do Paraná, Curitiba , PR – Brasil; 6 São PauloSP Brasil Hospital das Clínicas da Faculdade de Medicina da Universidade de São Paulo, São Paulo , SP – Brasil; 7 São José dos CamposSP Brasil Faculdade de Ciências Médicas de São José dos Campos – HUMANITAS, São José dos Campos , SP – Brasil; 8 Pouso AlegreMG Brasil Universidade do Vale do Sapucaí, Pouso Alegre , MG – Brasil; 9 Hospital das Clínicas Faculdade de São PauloSP Brasil Instituto da Criança e do Adolescente do Hospital das Clínicas da Faculdade de Medicina da Universidade de São Paulo, São Paulo , SP – Brasil; 10 São PauloSP Brasil Hospital Sancta Maggiore, São Paulo , SP – Brasil; 11 São PauloSP Brasil Faculdade de Medicina da Universidade de São Paulo, São Paulo , SP – Brasil; 12 São PauloSP Brasil Instituto do Câncer do Estado de São Paulo, São Paulo , SP – Brasil; 13 João PessoaPB Brasil Hospital Alberto Urquiza Wanderley, João Pessoa , PB – Brasil

## Abstract

Care for patients with cardiac arrest in the context of the coronavirus disease 2019 (COVID-19) pandemic has several unique aspects that warrant particular attention. This joint position statement by the Brazilian Association of Emergency Medicine (ABRAMEDE), Brazilian Society of Cardiology (SBC), Brazilian Association of Intensive Care Medicine (AMIB), and Brazilian Society of Anesthesiology (SBA), all official societies representing the corresponding medical specialties affiliated with the Brazilian Medical Association (AMB), provides recommendations to guide health care workers in the current context of limited robust evidence, aiming to maximize the protection of staff and patients alike.

It is essential that full aerosol precautions, which include wearing appropriate personal protective equipment, be followed during resuscitation. It is also imperative that potential causes of cardiac arrest of particular interest in this patient population, especially hypoxia, cardiac arrhythmias associated with QT prolongation, and myocarditis, be considered and addressed. An advanced invasive airway device should be placed early. Use of HEPA filters at the bag-valve interface is mandatory. Management of cardiac arrest occurring during mechanical ventilation or during prone positioning demands particular ventilator settings and rescuer positioning for chest compressions which deviate from standard cardiopulmonary resuscitation techniques. Apart from these logistical issues, care should otherwise follow national and international protocols and guidelines, namely the 2015 International Liaison Committee on Resuscitation (ILCOR) and 2019 American Heart Association (AHA) guidelines and the 2019 Update to the Brazilian Society of Cardiology Cardiopulmonary Resuscitation and Emergency Cardiovascular Care Guideline.

**Table:** 

Declaration of potential conflict of interests of authors/collaborators of the Position Statement: Cardiopulmonary Resuscitation of Patients with Confirmed or Suspected COVID-19 – 2020							
If, within the last 3 years, the author/collaborator of the statement:							
Names of statement collaborators	Participated in clinical and/or experimental studies sponsored by pharmaceutical or equipment companies related to this guideline	Spoke at events or activities sponsored by industry related to this guideline	Was (is) a member of a board of advisors or a board of directors of a pharmaceutical or equipment industry	Participated in normative committees of scientific research sponsored by industry	Received personal or institutional funding from industry	Wrote scientific papers in journals sponsored by industry	Owns stocks in industry
Álvaro Rea Neto	No	No	No	No	No	No	No
Ana Paula Freitas	No	No	No	No	No	No	No
Antonio Carlos Aguiar Brandão	No	No	No	No	No	No	No
Daniel Ujakow Correa Schubert	No	No	No	No	No	No	No
Fernando Bacal	No	No	No	No	No	No	No
Hélio Penna Guimarães	No	No	No	No	No	No	No
Ludhmila Abrahão Hajjar	No	No	No	No	No	No	No
Marcelo Antônio Cartaxo Queiroga Lopes	No	No	No	No	No	No	No
Matheus Fachini Vane	No	No	No	No	No	No	No
Natali Schiavo Giannetti	No	No	No	No	No	No	No
Roseny dos Reis Rodrigues	No	CSL Behring, Baxter, União Química, Octapharma	Halexistar	No	No	Octapharma	No
Sérgio Timerman	No	No	No	No	No	No	No
Thatiane Facholi Polastri	No	No	No	No	No	No	No
Thiago Domingos Correa	No	No	No	No	No	No	No
Thiago Timerman	No	No	No	No	No	No	No
Thomaz Bittencourt Couto	No	No	No	No	No	No	No

## 1. Introduction

Cardiopulmonary resuscitation (CPR) is perhaps the most extreme emergency procedure that can be required in a patient with coronavirus disease 2019 (COVID-19). In this setting, special caution is warranted, particularly regarding the increased risk of aerosol generation during chest compressions and ventilation, which poses a substantial hazard of rescuer contamination.

Considering the lack or inaccessibility of robust evidence on best practices in this novel scenario, the Brazilian Association of Emergency Medicine (ABRAMEDE), Brazilian Society of Cardiology (SBC), Brazilian Association of Intensive Care Medicine (AMIB), and Brazilian Society of Anesthesiology (SBA), all official societies representing the corresponding medical specialties affiliated with the Brazilian Medical Association (AMB), have issued this position statement containing specific recommendations for the management of cardiac arrest in patients with confirmed or suspected COVID-19. In all other cases, the 2015 guidelines of the International Alliance of Resuscitation Committees (ILCOR), the 2019 American Heart Association (AHA) guidelines,^1^ and the 2019 Update to the Brazilian Society of Cardiology Cardiopulmonary Resuscitation and Emergency Cardiovascular Care Guideline^2^ apply.

## 2. Prevention of Cardiac Arrest

- All patients with suspected or confirmed COVID-19 who are at increased risk of acute deterioration or cardiac arrest should be appropriately flagged to the local rapid response team (RRT) or whichever other team has been designated to provide code response.^3 - 5^ The use of severity scores and tracking systems, as well as the use of a “code yellow” system for identification of patients who are periarrest, allow early detection of critically ill patients and can optimize the care of cardiac arrest when it does occur;^2 , 5^
- Assessment of the potential difficulty of laryngoscopy/intubation must be performed on admission to the hospital and/or Intensive Care Unit (ICU) and recorded appropriately in the patient’s medical record. Scores such as MACOCHA ( *Mallampati, obstructive Apnea syndrome, reduced Cervical mobility, limited mouth Opening, Coma, severe Hypoxemia, and non-Anesthesiologist operator* ) or mnemonics such as LEMON ( *Look, Evaluate, Mallampati, Obstruction, Neck* ) can assist in determination of the difficult airway, activation of appropriate support, and prompt a request for a difficult airway trolley or cart;^6 , 7^
- Considering that two therapies currently under evaluation as potential treatments for COVID-19, chloroquine and hydroxychloroquine, may prolong the QT interval in up to 17% of patients, it is essential to consider the risk of severe polymorphic ventricular arrhythmias – especially *torsades de pointes* – and consequent occurrence of cardiac arrest with shockable rhythms;^4 , 8 - 10^
- The patients most at risk of polymorphic tachycardias in this context are older adults; women; and those with COVID-related myocarditis, heart failure, liver or kidney dysfunction, electrolyte disturbances (particularly hypokalemia and hypomagnesemia), and bradycardia. Identification of patients who already have a prolonged (> 500 ms) corrected QT interval (QTc) at baseline is paramount, and ECG monitoring should be performed daily as long as QT-prolonging drugs are used.^4 , 8 - 10^

## 3. Decision-making

- The decision of whether or not to initiate CPR must continue to be made on an individualized basis, be it during prehospital care, in the emergency department, or in the ICU. The potential benefits for the patient, the safety and exposure hazards of the code team, and the potential for futility of resuscitation maneuvers must be taken into account. Nevertheless, CPR should always be performed unless advance directives clearly state otherwise;^1 , 2^
- “Do not attempt CPR” (DNACPR) or “not for CPR” decisions/directives must be properly documented and communicated to the team. Palliative and end-of-life care should follow local and institutional policy.^1 , 2^

## 4. Guidance on Precautions

- Standard + aerosol precautions are recommended for all members of the code team in order to ensure adequate personal protection. Prompt availability of Personal Protective Equipment (PPE), e.g., by keeping PPE kits ready in every crash cart or trolley, minimizes the delay in initiating chest compressions and helps maintain continuity of care.^3 , 4 , 11 - 14^ Each PPE kit must include an N95 filtering facepiece respirator, face shield, waterproof gown, cap, long-cuff disposable gloves, and goggles;
- The safety of the code team should be the utmost priority even if this means delaying chest compressions, and all those who respond to the code must first don appropriate PPE. In particular, CPR should not be started on any patient with suspected or confirmed COVID-19 until the code team is fully attired with appropriate PPE;^3 , 4 , 11 - 14^
- The number of team members at the site of the code (if it is an enclosed space such as a private room or cubicle) should be restricted;^2 , 4 , 15 , 16^
- Hand hygiene plays an important role in reducing the transmission of COVID-19. All team members must wash their hands with soap and water (only when visibly soiled) or use an alcohol-based hand sanitizer;^3 , 15^
- Adherence to all applicable federal (Ministry of Health) and local government guidelines is mandatory.

## 5. First Response

- Recognition of cardiac arrest should follow ILCOR/AHA and Brazilian Society of Cardiology guidelines. Assessment should start by checking for responsiveness, breathing (chest rise and fall), and presence of a central pulse;^1 , 2^
- In adults, CPR should begin with continuous chest compressions. If the patient does not already have an invasive or advanced airway (orotracheal tube or extraglottic airway device) in place, a mask delivering low-flow oxygen or a towel should be placed over the patient’s mouth and nose before initiating compressions and kept in place until an invasive airway is secured,^8^ as chest compressions can generate aerosols;
- In children, CPR should preferably consist of compressions and ventilation with a bag-valve-mask (BVM) coupled to a high-efficiency particulate arrestance (HEPA) filter until a definitive airway is established, since pediatric arrest is most commonly of respiratory etiology, and compression-only CPR is known to be less effective in this population.^3^ If a BVM with HEPA filter is not available, compression-only CPR with a standard oxygen mask or towel covering the patient’s mouth is a reasonable alternative;^17^
- Despite the guidance of some emergency medical services that prehospital care of cardiac arrest in the absence of a medical professional (lay rescuer CPR) should be limited to hands-only CPR, the recommendation that the patient’s oral cavity be sealed to prevent aerosol generation as described above still stands;^4 , 8 , 9 , 14^
- Cardiac monitoring should be placed as soon as possible to ascertain whether there is a shockable rhythm, so as not to delay defibrillation if appropriate and provide guidance as to the optimal resuscitation algorithm to follow;^1 , 2^
- Defibrillation of a shockable rhythm should never be delayed to secure the airway or for other procedures;^1 , 2^
- If the patient already had a face mask in place to deliver supplemental oxygen before cardiac arrest occurred, it should be kept on until intubation, but delivering low-flow oxygen only (6–10 L/min at most); higher flow rates may be aerosol-generating;
- If the patient does not have any airway device in place, the rescuer should place a cloth or towel over the patient’s mouth and nose and begin continuous compressions;
- Before considering termination of CPR, any reversible causes should be identified and addressed, with particular emphasis on hypoxia, acidemia, and coronary thrombosis – all cited as common causes of death in recent publications on COVID-19.^3^ Additionally, polymorphic *torsades de pointes* -type ventricular tachycardia (associated with QT prolongation, which is known to be caused by drugs under investigation as potential COVID-19 treatments) and cardiac tamponade (associated with myocarditis), as well as ventilation-induced pneumothorax, have all been described as causes of cardiac arrest.

## 6. Airway Management

- BVM or bag-valve-tube (BVT) ventilation should be avoided, due to the high risk of aerosol generation and staff contamination.^3 , 15 , 18 , 19^ If BVM ventilation is absolutely necessary, two rescuers should always be present to allow a two-handed mask seal, and an oropharyngeal (Guedel) cannula should be placed. In this case, 30 compressions and two breaths should be performed in adults and 15 compressions and two breaths in children until an invasive airway has been established, at which point the ratio should switch to continuous compressions and one breath every 6 seconds for adults and children alike. Placement of a HEPA filter between the mask and the bag is recommended ( Figures 1 to 3 );
  **Figure 1:** – Bag-valve-mask device fitted with HEPA filter. Source: Personal collection.
- Considering that hypoxia is one of the main causes of cardiac arrest in patients with COVID-19, invasive airway access should be prioritized for isolation purposes, due to the lower likelihood of aerosol generation and, consequently, staff contamination, as well as the possibility of achieving better ventilation and oxygenation patterns.^15 , 16 , 19 - 21^ During airway instrumentation, chest compressions should be halted to protect the code team. It is suggested that airway instrumentation be performed or attempted during pulse checks, to reduce hands-off time. It is recommended that orotracheal intubation always be performed by the most experienced operator present;
- Videolaryngoscopy with a blade capable of providing a wide-angle view should be the first-line method of choice for quick, safe, and definitive airway management, ideally on first attempt, and always performed by the most experienced physician. In the event of intubation failure, the assistance of a second operator must be requested immediately. Videolaryngoscopy should again be prioritized for the second attempt;^16 , 20 , 21^
- For children, videolaryngoscopy with a blade suitable for the size of the patient is recommended; there is no particular need for a wider view angle;^20^
- If intubation fails again or is deemed impossible, an extraglottic device (laryngeal tube or laryngeal mask) should be placed. This will allow closed-circuit mechanical ventilation and capnography until conditions are present for establishment of a definitive (surgical) airway via tracheostomy or cricothyrotomy.^20 , 22^ In children, a laryngeal mask suitable for the patient’s weight and size is the extraglottic device of choice.^23^ In Brazil, placement of extraglottic airway devices is within the scope of practice of both physicians and nurses, and can thus be an alternative for airway management in prehospital intermediate life support or limited advanced life support, as well as in nurse-led codes.^1 , 2^ Nevertheless, endotracheal intubation is still recommended whenever possible, largely with the aim of reducing aerosol generation;
- When more than one extraglottic device is available, priority should be given whenever possible to that providing the best airway seal and the possibility of sequential placement of an orotracheal tube through the device lumen (Fastrach™ or other intubating laryngeal mask airway);
- Even after a patient has been intubated or has an extraglottic device in place, occlusion and sealing of the oral cavity is still important to reduce aerosolization; this can be done with towels, gauze packs, or a standard surgical mask;
- When cardiac arrest occurs in a patient already on mechanical ventilation, the patient should be connected to the ventilator through a closed ventilation circuit and the ventilator parameters set as follows ( Chart 1 ):
  **Table:** Chart 1 – Mechanical ventilator settings for cardiopulmonary resuscitation.

Volume assist-control ventilation mode; VT = 6 mL/kg PBW Respiratory rate = 10 bpm FiO2 = 100% Flow trigger = disabled or sensitivity threshold -15 to -20 PEEP = 0 VT alarms = maximum and minimum allowed by device Pressure alarms = 60 cmH_2_O maximum, 1 or 0 cmH_2_O minimum High and low minute volume alarms = maximum and minimum allowed by device Apnea time = 60 seconds

FiO_2_: fraction of inspired oxygen; PBW: predicted body weight; PEEP: positive end-expiratory pressure; VT: tidal volume.

Source: Personal collection.
  ○ Mode: volume assist-control ventilation (AC or ACV). Tidal volume (V_T_): 6 mL/kg predicted body weight.
  ○ Fraction of inspired oxygen (FiO_2_): 100%.
  ○ Respiratory rate (RR): approximately 10 breaths per minute; inspiratory time (Ti): 1 second.
  ○ Flow triggering: off; if triggering cannot be disabled, switch to pressure-triggering mode and adjust the triggering pressure to the least sensitive (i.e., lowest) possible threshold; this ranges from -15 to -20 depending on ventilator model.
  ○ Positive end-expiratory pressure (PEEP): zero.
  ○ Alarms: set tidal volume alarms to the minimum and maximum allowed by the ventilator and pressure alarms to 60 cmH_2_O (maximum) and 1 or 0 cmH_2_O (minimum). The high and low minute volume alarms should be set to the maximum and minimum allowed by the device. The respiratory rate alarm should be set to the maximum allowed by the device, and the apnea time, to 60 seconds.

- The exact same parameters apply in children;

Continuously assess whether the ventilator can maintain these parameters without auto-triggering, which leads to hyperventilation and air trapping with excessive pressures (systematically above 60 cm H_2_O). In children, temporary disconnection from the ventilator may be necessary; in this case, BVM ventilation with a HEPA filter should be performed;

- Some ventilators provide a “CPR mode” function, which automatically adjusts the alarm limits and ventilator parameters as described above. For mechanically ventilated patients, it is recommended that a HEPA filter be placed in the ventilation circuit after the orotracheal tube and a second filter at the expiratory circuit;^16 , 20 , 21^
- To minimize aerosol generation, the tube should be clamped with a strong straight hemostat whenever there is a need to switch ventilator circuits (BVM to mechanical ventilator circuit, for instance);
- For the safety of the team and the patient, adhesive pads (which do not require disconnection from the ventilator) should always be preferred for defibrillation. If manual (paddle) defibrillation is needed, the ventilator should be placed in standby mode and the orotracheal tube disconnected from the ventilator, keeping the HEPA filter attached to the tube, only after the shock has been delivered.

## 7. Chest Compressions

- High-quality chest compressions should be performed, ensuring:
  ○ A compression rate of 100 to 120 compressions per minute.
  ○ In adults, a compression depth of at least 5 cm (compressions deeper than 6 cm should be avoided).
  ○ In infants, compression depth should be one-third of the anteroposterior diameter of the chest; in children, it should be one-third of the anteroposterior diameter of the chest or at least 5 cm.

- Allow full recoil of chest after each compression; do not lean on the patient’s chest;
- Minimize interruptions in chest compressions; pauses should be limited to 10 seconds at most (for two breaths). Consider performing CPR with the goal of the highest possible chest compression fraction, aiming at a minimum of 60% to 80%;
- Rotate out with another team member every 2 minutes to avoid rescuer fatigue, which can lead to poor compressions;
- If the patient is in the supine position, compressions should be performed in the center of the chest, on the lower half of the breastbone (sternum);
- Considering the need for PPE use to limit the risk of aerosol generation, the strenuous nature of resuscitation maneuvers, the potential for rescuer fatigue and exhaustion, and the need to minimize the number of team members present during resuscitation, use of a mechanical chest compression device is advised for adults whenever one is available.

## 8. Cardiopulmonary Resuscitation in the Prone Position

- If the patient is in prone position with no invasive airway in place, he or she should be quickly repositioned supine, CPR should be initiated, and an invasive airway device should be placed as soon as possible, preferably by orotracheal intubation;
- If the patient is already intubated and ventilated, it is recommended that CPR maneuvers be initiated with the patient still in prone position. The surface landmark for hand placement is the exact posterior projection of the site for chest compressions, i.e., in the interscapular region, at the T7-T10 level ( Figure 4 ). Attempts to de-prone (i.e., return the patient to the supine position) should be performed with maximum care to avoid ventilator disconnection and minimize the risk of aerosolization. If adhesive defibrillator pads are available, they should be placed in an anteroposterior arrangement;^10 , 22 , 23^
  **Figure 4:** – Hand placement for compressions on a patient in the prone position. 23
- If no adhesive pads are available, manual defibrillation can be attempted by placing the sternal paddle on the dorsal region and the apical paddle on the patient’s flank ( Figure 5 ). It is recommended that the effectiveness of CPR be assessed by end-tidal CO_2_monitoring (partial pressure of carbon dioxide > 10 mmHg) and invasive blood pressure monitoring (diastolic blood pressure > 20 mmHg). It bears stressing that evidence for this maneuver is still unclear and, whenever possible, the patient should be de-proned, as the supine position is best suited for high-quality CPR and adequate ventilation.
  **Figure 5:** – Suggested paddle position for manual defibrillation of a patient in the prone position. 23

## 9. Post-cardiac Arrest Care

- If the patient is not already in intensive care, an ICU bed with respiratory isolation should be requested even before return of spontaneous circulation (ROSC) is achieved;^3 , 15 , 16^
- All equipment used during CPR should be disposed of or sanitized following manufacturer recommendations and institutional or local guidelines;^3^
- All surfaces onto which airway/resuscitation equipment was placed must also be cleaned as per local guidelines. Check that no airway management devices (including laryngoscopes and face masks) have been left on the bed. All equipment should be left on the intubation tray if possible;^3^
- After the code, team members should doff all PPE safely, avoiding self-contamination.^3 , 15^ Particular attention is required during this step, which is when contamination of health care workers is most likely to occur (through contact with patient secretions and respiratory droplets).

## 10. Specific Guidance for Prehospital Care

- In the prehospital environment, CPR should never be attempted in patients with suspected or confirmed COVID-19 who present with obvious signs of death;^3^
- Prehospital care providers should follow standard + aerosol precautions when caring for patients with suspected or confirmed COVID-19;
- The population should be instructed to notify the dispatcher if the victim is suspected to have COVID-19 when calling an ambulance. This allows prehospital care providers to don appropriate PPE before arrival at the scene. Emergency medical service dispatchers and physician regulators/medical directors are strongly advised to conduct active case-finding of COVID-19 by inquiring about flu-like symptoms, fever, and dyspnea during calls;
- CPR should be limited to continuous chest compressions. Mouth-to-mouth ventilation, even with use of a pocket CPR mask, should never be performed for patients with suspected or confirmed COVID-19;^3^
- Considering that most out-of-hospital cardiac arrests occur at home, in pediatric out-of-hospital arrests, the lay rescuer will most likely be a parent, family member, or caregiver who will already be in close contact with the child and thus exposed to respiratory secretions. In this case, the lay rescuer should be instructed to perform compressions and consider mouth-to-mouth ventilation if he or she is able and willing to do so, since most pediatric arrests are secondary to respiratory causes;^23^
- Hands-only CPR is a reasonable alternative if the rescuer is unable or unwilling to provide mouth-to-mouth resuscitation or has not had close contact with the child before;^17^
- The rescuer should cover the victim’s mouth and nose with a cloth or towel (or, if available, place a mask delivering low-flow oxygen) to prevent suspension of aerosols generated during CPR;
- Do not delay defibrillation. Early use of an automated external defibrillator (AED) significantly increases the odds of survival and does not increase the risk of COVID-19 transmission;
- Positive-pressure BVM ventilation should be avoided at all cost. If absolutely necessary, it must always be performed by two providers, one of whom will be exclusively responsible for sealing the mask to the patient’s face, using the most suitable grip technique to avoid air leak. A BVM may only be used if a HEPA filter is available and has been placed at the bag-valve interface.
- In children, CPR should preferably consist of chest compressions and BVM ventilation (always with a HEPA filter);
- Otherwise, prehospital airway management should follow the aforementioned recommendations for in-hospital care – namely, ensuring that a BVM and any other ventilation devices are equipped with HEPA filters and that an advanced airway device (tracheal tube or extraglottic airway) is placed as early as possible;
- Open the rear doors of the transport vehicle and activate the heating, ventilation, and air conditioning (HVAC) system during any aerosol-generating procedures (do this away from pedestrian traffic);
- Family members or chaperones may not ride along in the ambulance in the same compartment as the patient. According to Ministry of Health recommendations, patients with suspected or confirmed COVID-19 are not allowed any chaperones who may be at risk of contamination. It is suggested that companions or chaperones be instructed to make their own way to the health facility;
- If the transport vehicle lacks an isolated driver compartment, the outside air vents in the driver’s area should be opened and the rear exhaust fans turned on at the highest setting.

## 11. Training and Debriefing

- Perform debriefing at the end of each code to support team growth and improvement;^1 , 2^
- All health care workers involved in the care of patients with suspected or confirmed COVID-19 should undergo skills training in donning and, especially, doffing PPE as soon as possible, as well as participate in simulated code blue response;^15 , 16 , 20 , 21^
- Skills training and continuing medical education are paramount to protecting staff and improving safety in patient care. The use of moulages/vignettes, realistic simulation resources, and distance education resources is strongly recommended.

Following are the algorithms for PCR care of adult ( Figure 6 ) and pediatric ( Figure 7 ) patients with suspected or confirmed COVID-19.

**Figure 6:** – Algorithm for management of cardiopulmonary arrest in patients with suspected or confirmed COVID-19. AMI: acute myocardial infarction; BP: blood pressure; BVM: bag-valve-mask; CPR: cardiopulmonary resuscitation; Fi: inspired fraction; HEPA: high-efficiency particulate arrestance; IO: intraosseous; IV: intravenous; MV: mechanical ventilation; PE: pulmonary embolism; PEA: pulseless electrical activity; PEEP: positive end-expiratory pressure; ROSC: return of spontaneous circulation; RR: respiratory rate; Ti: inspiratory time; VF: ventricular fibrillation; VT: ventricular tachycardia.

**Figure 7:** – Algorithm for management of cardiopulmonary arrest in pediatric patients with suspected or confirmed COVID-19. AMI: acute myocardial infarction; BP: blood pressure; BVM: bag-valve-mask; CPR: cardiopulmonary resuscitation; Fi: inspired fraction; HEPA: high-efficiency particulate arrestance; IO: intraosseous; IV: intravenous; MV: mechanical ventilation; PE: pulmonary embolism; PEA: pulseless electrical activity; PEEP: positive end-expiratory pressure; ROSC: return of spontaneous circulation; RR: respiratory rate; Ti: inspiratory time; VF: ventricular fibrillation; VT: ventricular tachycardia.

**Figure 2:** – Manikin simulation of an intubated patient being ventilated with a bag-valve-mask device fitted with a HEPA filter. Note surgical mask covering the nose and oral cavity. Source: Personal collection.

**Figure 3:** – Manikin simulation of a patient with an extraglottic airway device being ventilated. Note HEPA filter and surgical mask covering the nose and oral cavity. Source: Personal collection.
